# Disturbed microbiota-metabolites-immune interaction network is associated with olfactory dysfunction in patients with chronic rhinosinusitis

**DOI:** 10.3389/fimmu.2023.1159112

**Published:** 2023-05-23

**Authors:** Xingyu Han, Xuejia He, Xiaojun Zhan, Linyin Yao, Zhifu Sun, Xing Gao, Shan Wang, Zhenlin Wang

**Affiliations:** ^1^ Department of Otolaryngology-Head and Neck Surgery, Xuanwu Hospital, Capital Medical University, Beijing, China; ^2^ Department of Otolaryngology-Head and Neck Surgery, Beijing Anzhen Hospital, Capital Medical University, Beijing, China; ^3^ Department of Otorhinolaryngology-Head and Neck Surgery, Capital Institute of Pediatrics, Beijing, China; ^4^ Capital Institute of Pediatrics, Peking University Teaching Hospital, Beijing, China; ^5^ Beijing Municipal Key Laboratory of Child Development and Nutriomics, Capital Institute of Pediatrics, Beijing, China

**Keywords:** olfactory dysfunction, inflammatory cytokines, type 1/type 2 immune response, chronic rhinosinusitis, metagenomics, metabolomics

## Abstract

**Purpose:**

Olfactory dysfunction (OD) is a debilitating symptom frequently reported by patients with chronic rhinosinusitis (CRS) and it is associated with a dysregulated sinonasal inflammation. However, little information is available about the effect of the inflammation-related nasal microbiota and related metabolites on the olfactory function in these patients. Therefore, the current study aimed to investigate the nasal microbiota-metabolites-immune interactions and their role in the pathogenesis of OD in CRS patients.

**Methods:**

23 and 19 CRS patients with and without OD, respectively, were enrolled in the present study. The “Sniffin’ Sticks” was used to measure the olfactory function, while the metagenomic shotgun sequencing and the untargeted metabolite profiling were performed to assess the differences in terms of the nasal microbiome and metabolome between the two groups. The levels of nasal mucus inflammatory mediators were investigated by a multiplex flow Cytometric Bead Array (CBA).

**Results:**

A decreased diversity in the nasal microbiome from the OD group compared to the NOD group was evidenced. The metagenomic analysis revealed a significant enrichment of *Acinetobacter johnsonii* in the OD group, while *Mycoplasma arginini*, *Aeromonas dhakensis*, and *Salmonella enterica* were significantly less represented (LDA value > 3, p < 0.05). The nasal metabolome profiles were significantly different between the OD and NOD groups (*P* < 0.05). The purine metabolism was the most significantly enriched metabolic subpathway in OD patients compared with NOD patients (*P* < 0.001). The expressions of IL-5, IL-8, MIP-1α, MCP-1, and TNF were statistically and significantly increased in the OD group (*P* < 0.05). All these data, including the dysregulation of the nasal microbiota, differential metabolites, and elevated inflammatory mediators in OD patients demonstrated a clear interaction relationship.

**Conclusion:**

The disturbed nasal microbiota-metabolite-immune interaction networks may be implicated in the pathogenesis of OD in CRS patients and the underlying pathophysiological mechanisms need to be further investigated in future studies.

## Graphical Abstract

This study demonstrated alterations in the nasal metagenome, metabolome, and inflammatory mediators of nasal mucus in chronic rhinosinusitis (CRS) patients with olfactory dysfunction (OD) compared to patients with non-olfactory dysfunction (NOD). Integrated multi-omics analysis revealed nasal microbiota-metabolites-immune interaction networks perturbed in OD patients. The picture was created with BioRender.com.

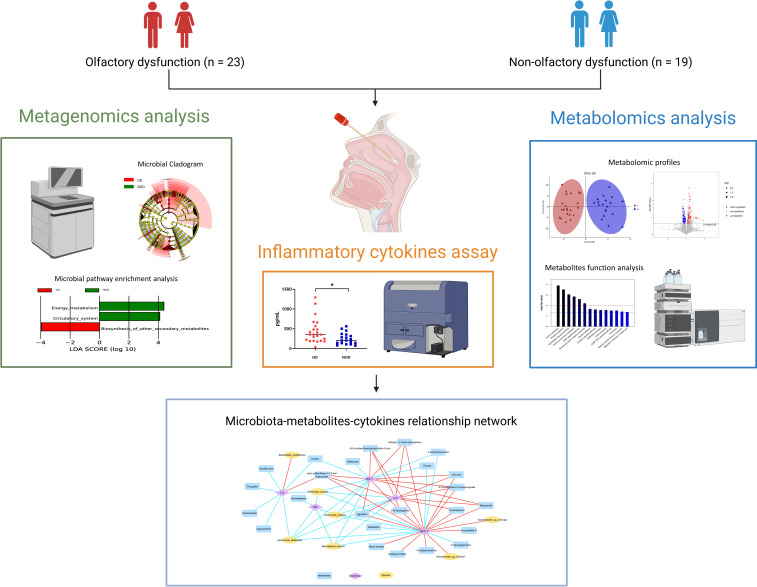

## 1 Introduction

Chronic rhinosinusitis (CRS) represents one of the most common chronic diseases affecting worldwide 12% of people (1). CRS may lead to a severe adverse impact on quality of life (QOL) and a significant increase in healthcare expenses. Olfactory dysfunction (OD) is a crucial symptom of CRS affecting up to 60-80% of CRS patients and gives a great contribution to the decreased QOL in these patients (2). On the other side, CRS is also the leading cause of olfactory disorders, accounting for 14-30% of the total. Given that the olfactory ability is responsible for the moderation of nutrient intake, prevention of environmental hazards, and reflection of interpersonal communication information, it is deductible that the related impairment may have severe consequences. In addition, a reciprocal relationship between olfaction and depression has been demonstrated considering the shared neural processing pathways, and OD is often accompanied by depression symptoms (3). Furthermore, epidemiological studies showed that OD is an independent predictor of 5-year mortality (4, 5).

Despite the high incidence and serious impact of OD in CRS, the underlying pathogenesis remains unknown. The current research stated that the chronic inflammation of the olfactory neuroepithelium is the main cause of CRS-related OD (6). Specifically, olfactory inflammation could induce the release of neurotoxic inflammatory mediators while disrupting the odor signal communication between the mucous layer and olfactory epithelium (7). Additionally, the imbalance of type 1/type 2 immune responses plays a crucial role in the pathophysiology of OD in CRS patients (8–11). The excessive type 2 inflammation response, which is characterized by enhanced eosinophilia infiltration and increased type 2 cytokines such as IL-4, IL-5, and IL-13, could lead to a release of eosinophilic-derived neurotoxin, resulting in olfactory sensory neuron apoptosis and restrain neural regeneration (8, 12). Similarly, a persistent chronic type 1 inflammation in CRS, mediated by TNF (Tumor Necrosis Factor) and IFN-γ (Interferon-γ), could determine a switching of the olfactory stem cells’ function from regeneration to immune defense (9).

In addition, the nasal microbiota is considered the main environmental driver of the inflammatory process in CRS (13) as the dysfunctional interaction between microorganisms and the host immune system triggers mucosal inflammation (1). Indeed, dysbiosis of the nasal flora can disrupt the barrier integrity and induce an increased invasion of pathogenic bacteria, further contributing to CRS. Recent reports demonstrated that *S. aureus* is mostly associated with CRS, and it drives the type 2 inflammatory responses through the secretion of enterotoxins or by binding to Toll-like receptor 2 (TLR2) (14, 15). Additionally, *Haemophilus* and *Streptococcus* may be involved in the recruitment of neutrophils and release of IL-8 in non-type 2 CRS (16, 17). However, the exact role of the nasal microbial community in the pathogenesis of CRS-related OD remained unknown.

Furthermore, the interaction between metabolic disorders and inflammatory responses in CRS patients has received increasing attention in recent decades. Indeed, the dysregulated fatty acid metabolism was observed in CRS and it appears associated with elevated type 2 cytokines and tissue eosinophils (18, 19), and the level of eicosanoid was reported to be significantly correlated with clinical disease severity in CRS patients (20). Besides, some protective metabolites, such as 12/15-LOX-derived lipid mediators, could contribute to the resolution of airway inflammation (21). However, the effect of altered metabolites on the olfactory function in CRS patients should still be investigated.

Therefore, in this study, it was hypothesized that both nasal microbial community dysregulation and metabolic dysfunction may play a role in the pathogenesis of OD in CRS patients by modulating the inflammatory response of the olfactory mucosa. We aimed to (1) examine the alterations of the microbiota composition, metabolic products, and inflammatory mediators in nasal mucus samples from OD patients (2); investigate the role of the nasal microbiota-metabolites-immune interaction in relation to OD in CRS patients.

## 2 Material and methods

### 2.1 Study participants

In total, 23 CRS patients with OD and 19 CRS patients of the same age and sex without OD (NOD) admitted to the otorhinolaryngology Department of Beijing Anzhen Hospital were enrolled in this study. All participants matched the diagnostic criteria for chronic rhinosinusitis according to the European Position Paper on Rhinosinusitis and Nasal Polyps of 2020 (1). The exclusion criteria adopted included: < 18 years old; presence of other types of olfactory disorders; previous nasal surgery; concomitant presence of an autoimmune or immunodeficiency disease; treatment with antibiotics, corticosteroid, antihistamines, and leukotrienes receptor antagonists in the month before the sampling; presence of an acute exacerbation of chronic rhinosinusitis. The study design was approved by the Ethics Committee at Beijing Anzhen Hospital (GZR-3-077). All the subjects agreed with written informed consent before enrolling in the study.

### 2.2 Clinical assessment

Based on the nasal endoscopy exam, patients were classified into chronic rhinosinusitis with nasal polyposis (CRSwNP) and chronic rhinosinusitis without nasal polyposis (CRSsNP). The 22-item Sino-Nasal Outcome Test (SNOT-22) was used to assess the CRS-related quality of life (22). The radiological and endoscopic severity of the sinus disease was assessed by the Lund-Mackay scoring system and the Lund-Kennedy scale, respectively (23, 24). Demographic and clinical data, including age, gender, smoking/drinking habits, asthma history, and related hematological indices, were collected from all the participants.

The olfaction function was assessed utilizing the authoritative and validated olfaction assessment tool—Sniffin’ sticks (25), which was composed of three subtests—odor threshold, odor discrimination, and odor identification. The odor threshold subtest was comprised of felt-tip pens with 16 different concentrations of phenethyl alcohol (PEA), and the participants were asked to discriminate the odor-containing pen from two blanks through a three-alternative forced choice (3-AFC) task (odor threshold score ranged from 1 to 16 points). The odor discrimination test consisted of 16 triplets of pens, with one of which contained a different odor than the other two. Participants were required to determine the specific pen with a different odor through a 3-AFC task. The number of correctly identified odors was an odor discrimination score ranging from 0 to 16 points. The odor identification test contained 16 different pens with common and familiar odorants. The subjects were required to identify and label the odor of the given pen under the prompt of the four alternative descriptors. The odor identification score corresponded to the number of correctly identified odors.

The sum of the three subtest scores was counted as the total TDI scores, with a range from 1 to 48 points. TDI scores ≥ 30.75 were considered indicative of a normal olfactory function. When the score was < 30.75, the OD was diagnosed. Of these, TDI scores between 16.25 and 30.5 were considered to be hyposmia (decreased olfactory function), while if below or equal to 16.0 they were considered to be functional anosmia (absent olfactory function).

### 2.3 Sample collection

Nasal mucus samples were collected on the same day of the olfactory function evaluation. Under endoscopic guidance, a 22 * 7 * 2 mm polyurethane sponge was positioned in the middle nasal meatus of the participants for a total of 10 minutes. After, the sponge was removed and immediately put into a microporous centrifugal filter device, followed by centrifugation at 14,000g for 15 min in order to elute mucus, which was placed into a new microcentrifuge tube and frozen at −80°C until use.

### 2.4 DNA extraction and metagenomic shotgun sequencing

DNA was extracted from nasal mucus samples through a MagPure Soil DNA KF Kit (Magen Biotechnology, Guangzhou, China). The DNA integrity and concentration were evaluated by an agarose gel electrophoresis and a NanoDrop 2000 spectrophotometer (Thermo Fisher Scientific, Waltham, Massachusetts, USA), respectively. DNA was then fragmented by S220 Focused-ultrasonicators (Covaris, Woburn, Massachusetts, USA) and cleaned up by Agencourt AMPure XP beads (Beckman Coulter Co., Indianapolis, Indiana, USA). Then the library construction was conducted by utilizing a TruSeq Nano DNA LT Sample Preparation Kit (Illumina, San Diego, California, USA) following the manufacturer’s instructions. A metagenomic sequencing approach was performed using an Illumina Novaseq 6000 platform (Illumina, San Diego, California, USA), and 150 bp paired-end reads were yielded.

### 2.5 Metagenomic bioinformatic analysis

Trimmomatic (v 0.36) was used to trim and filter raw data (26). The screened reads were assembled using MEGAHIT (v 1.1.2) (27). Prodigal (v 2.6.3) was used to predict ORFs which were translated into amino acid (aa) sequences (28). Non-redundant gene sets were clustered for all predicted genes setting an aa identity of 95% and a coverage of 90% using CDHIT (v 4.5.7), and the longest gene of each genome was selected as the representative sequence. Bowtie2 (v 2.2.9) was used to clean reads which were then compared to the non-redundant gene set (95% of aa identity) followed by the calculation of the abundant information for genes for each corresponding sample.

The taxonomy of the species was obtained using the taxonomy database of the NR Library of NCBI, while the abundance of the microbial species was determined according to the corresponding abundance of the genes. The gene set representative sequences were compared with the NR library using DIAMOND (v 0.9.7) software (29) to select the proteins with the highest sequence similarity and obtain functional annotation information.

Principal Coordinates Analysis (PCoA) was performed using R software (v 3.2.0) to assess the diversity in microbiota composition between OD and NOD groups. The univariate tests of the differences in nasal microbial β-diversity between the OD and NOD groups were conducted using a permutational multivariate analysis of variance (PERMANOVA) through the vegan package in R. Then the linear discriminant analysis effect size (LEfSe) was used to further compare the taxonomy abundance spectrum between different groups, and define the potential biomarkers of CRS-related OD (30).

### 2.6 Untargeted metabolite profiling by LC-MS and GC-MS

The untargeted metabolite profiling was conducted through liquid chromatography-mass spectrometry (LC-MS) and gas chromatography-mass spectrometry (GC-MS). Following a standard procedure, the samples were analyzed on ACQUITY UPLC I-Class system (Waters Corporation, Milford, Massachusetts, USA) together with Q-Exactive quadrupole-Orbitrap mass spectrometer (Thermo Fisher Scientific, Waltham, Massachusetts, USA) in both ESI+ and ESI− ion modes. Meanwhile, the Agilent 7890B gas chromatography system coupled to an Agilent 5977B MSD system (Agilent Technologies Inc., California, USA) was also employed in the metabolic profiling analysis. All the samples were extracted in equal volumes and mixed as quality control (QC) samples, which were intermittently inserted during the entire analysis to assess the reproducibility of the mass spectrometry system.

The LC-MS raw data were analyzed by the Progenesis QI v2.3 software (Nonlinear, Dynamics, Newcastle, UK) to collect data about peak intensity, the retention time of compounds, and the mass-to-charge ratio. A qualitative analysis of compounds was performed by using the Human Lipidmaps (v2.3), Metabolome Database (HMDB), Metlin database, PMDB, and EMDB databases (OE Biotech *in-house* developed databases) based on the precise mass-to-charge ratio (M/z), secondary fragments, and isotopic distribution. The positive and negative data were all combined into a total data matrix for subsequent analysis.

Original GC-MS data were formatted using the software Analysis Base File Converter and processed through the software MS-DIAL. Then LUG database was used for the metabolites characterization. All peak signal intensities in each sample were split and normalized according to a filtered internal criterion of RSD greater than 0.3. After data normalization, redundancy removal and peak merging were carried out to obtain the final data matrix.

The Orthogonal Partial Least-Squares-Discriminant Analysis (OPLS-DA) was employed to examine the metabolite differences between OD and NOD groups. The validity of the model was assessed using a 7-fold cross-validation and 200 response permutation tests (RPT) to avoid over-fitting. Based on OPLS-DA, differential metabolites were selected with Variable Importance of Projection (VIP) values > 1 and *P* values < 0.05.

### 2.7 Mucus cytokines assay

The levels of the nasal mucus inflammatory mediators, including IL-4, IL-5, IL-8, IL-10, IL-13, IP-10, IFN-γ, MIP-1α, Basic FGF, Eotaxin, RANTES, MCP-1, TNF, IgE, were investigated utilizing using a multiplex flow Cytometric Bead Array (CBA) Human Cytokine Kits (BD Biosciences, San Jose, California, USA). CBA assay was performed on FACSCanto-123 (BD Biosciences, San Jose, California, USA) according to the manufacturer’s protocol while the data analysis was conducted by BD FCAP Array Software version 3.0.

### 2.8 Statistical analysis

The statistical analyses for clinical data were performed using SPSS version 27.0 (SPSS, Chicago, Illinois, USA). Demographic and clinical data were reported as mean ± SD or median and interquartile range (IQR), and the differences between groups were analyzed by t-test or Mann-Whitney U test, respectively. Differences between groups in relation to all the categorical variables were analyzed by χ^2^ analysis. A two-sided *P* value < 0.05 was statistically significant. The R package (ggplot2) was used to analyze the significant differences in terms of taxonomy abundance between groups using the Metastats analysis (Wilcoxon statistical test) (31). To analyze the correlations between the variables, a Spearman correlation test was conducted using *Origin 2022* (OriginLab, Northampton, MA, USA).

## 3 Results

### 3.1 Demographics and clinical characteristics of study participants

The demographic and clinical characteristics of the participants are reported in **Table 1**. No statistically significant differences in terms of age and gender distribution (*P* > 0.05) was found between the two groups, as well as in terms of the proportion of different CRS phenotypes (*P* > 0.05). Additionally, no significant differences in the disease severity indicators of CRS were found between groups, including SNOT-22 scores, Lund-Kennedy endoscopy scores, and Lund-Mackay CT scores (*P* > 0.05). The TDI scores were significantly lower in the OD group than in the NOD group (*P* < 0.001). The blood eosinophil percentages in OD patients were significantly higher than in NOD patients (*P* = 0.049). The groups did not significantly differ in smoking and drinking habits (*P* > 0.05).

**Table 1:** Demographics and clinical data of participants enrolled in the study.

	OD group (n=23)	NOD group (n=19)	*P* value
Males, n [%]	16 [69.6%]	16 [84.2%]	0.267
Age, years	34.0 (29.6-52.0)	30.0 (27.6-34.0)	0.061
CRS phenotypes			0.453
CRSwNP, n [%]	17 [73.9%]	12 [63.2%]	
CRSsNP, n [%]	6 [26.1%]	7 [36.8%]	
SNOT-22 score	30.57 ± 16.28	35.58 ± 18.40	0.355
Lund-Kennedy endoscopy score	6.00 ± 1.95	5.18 ± 2.07	0.112
Lund-Mackay CT score	11.74 ± 5.42	10.12 ± 2.93	0.153
TDI scores	21.0 (12.0-23.5)	31.5 (31.5-33.0)	<0.001**
Blood eosinophil count (cells ×10^9^/L)	0.19 (0.10-0.33)	0.11 (0.02-0.25)	0.087
Blood eosinophil percentage (%)	3.60 (1.50-5.00)	1.50 (0.20-4.10)	0.049*
Blood basophil count (cells × 10^9^/L)	0.03 (0.02-0.04)	0.03 (0.02-0.06)	0.967
Blood basophil percentage (%)	0.60 (0.30-0.70)	0.40 (0.20-0.60)	0.247
Blood Neutrophil count (cells × 10^9^/L)	3.42 (2.86-4.21)	5.40 (3.02-6.74)	0.092
Blood Neutrophil percentage (%)	58.51 ± 9.64	59.65 ± 11.77	0.738
Asthma, n [%]	3 [13.0%]	1 [5.3%]	0.393
Smoker, n [%]	6 [26.1%]	4 [22.2%]	0.775
Drinker, n [%]	3 [13.0%]	4 [22.2%]	0.438

Results are expressed as mean ± SD, median (interquartile range), or n (%).

CRSwNP: chronic rhinosinusitis with polyps; CRSsNP: chronic rhinosinusitis without polyps.

* P < 0.05; ** P < 0.01.

### 3.2 Diversity and composition of the nasal microbiome between OD and NOD groups

**Figure 1:**
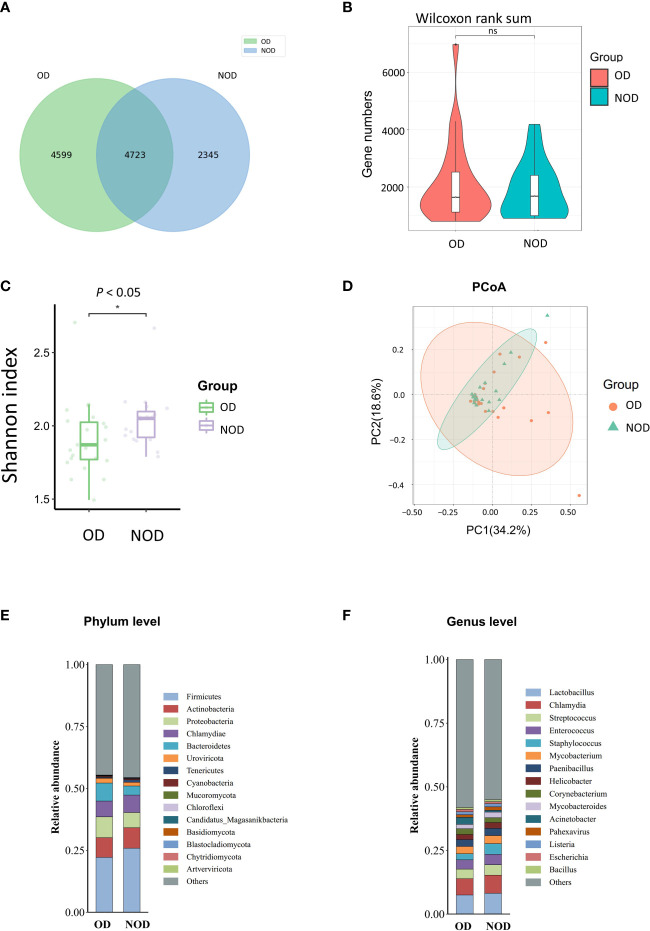
Nasal microbiota of OD (n = 23) and NOD patients (n = 19). **(A**, **B)** Gene number differences between groups. **(C)** Differences in Shannon α-diversity indices of microbial community between groups. **(D)** Principal coordinates analysis (PCoA) of the nasal microbiota in OD and NOD patients (*P* = 0.24). **(E**, **F)** Bar plots of the nasal microbiota composition at phylum and genus levels. * *P* < 0.05.

To investigate differences in nasal microbiota in OD patients, we applied a shotgun metagenomics approach, and 13,002 clean reads were obtained. Clean reads from each sample were compared to the non-redundant gene set to obtain information about the related gene abundance. The Venn diagram showed 4,599 unique genes in the OD group, 2,345 unique genes in the NOD group, and 4,723 shared by the two groups (**Figure 1A**). The difference in the observed gene number was not statistically significant by the violin plot (*P* > 0.05) (**Figure 1B**). The α-diversity analysis demonstrated that the Shannon diversity index in the OD group was significantly lower than that of the NOD group, indicating less microbial community diversity in the OD group (*P* < 0.05) (**Figure 1C**). Regarding the β-diversity, Principal Coordinates Analysis (PCoA) based on Bray-Curtis dissimilarity did not report any statistically significant difference in the nasal microbiota composition between the two groups at a species level (PERMANOVA, R2 = 0.029, *P* = 0.24) (**Figure 1D**).

Additionally, the composition of the nasal microbiome among groups was compared at a phylum and genera level (**Figures 1E, F**). *Firmicutes, Actinobacteria, Proteobacteria, Chlamydiae*, and *Bacteroidetes* were the predominant phyla among both groups, with *Bacteroidetes* significantly more elevated in the OD group than in the NOD group (*P* = 0.047). On the other hand, *Lactobacillus, Chlamydia, Streptococcus, Enterococcus*, and *Staphylococcus* were the predominant genera in both groups.

### 3.3 Nasal microbiome differences among the OD and NOD groups at a species level

**Figure 2:**
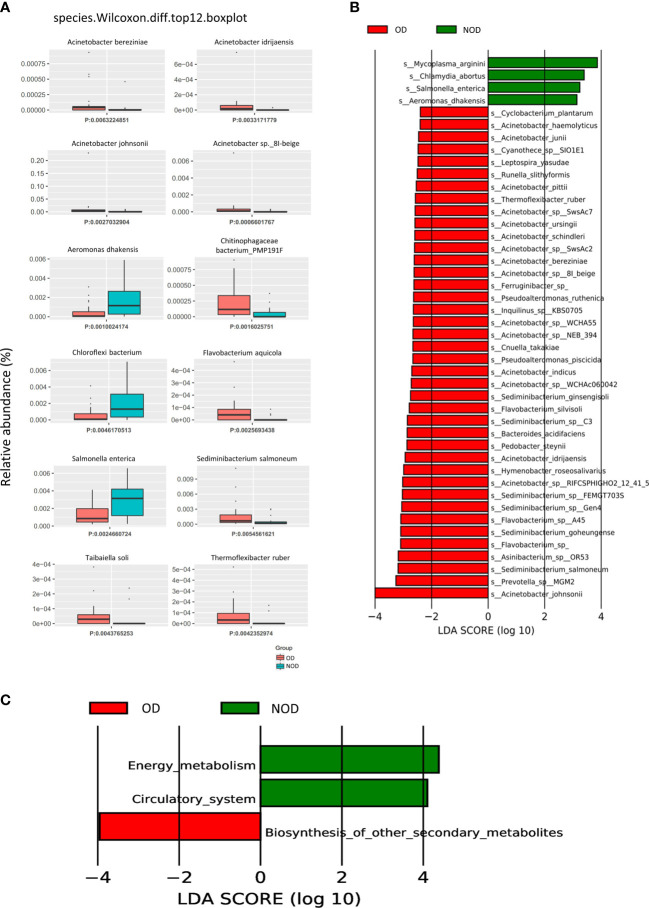
Difference in nasal microbial species between the OD (n = 23) and NOD groups (n = 19) and microbial gene function annotation. **(A)** Microbial species differences by Metastats analysis between OD and NOD groups. **(B)** Differential species based on Linear discriminant analysis (LDA) effect size (LEfSe) between OD and NOD groups. **(C)** Kyoto Encyclopedia of Genes and Genomes (KEGG) function differences of the nasal microbiota between OD and NOD groups.

The Metastats analysis (Wilcoxon rank-sum test) showed significant differences in the species composition between the OD and NOD groups (**Figure 2A** and **Supplementary Table S1**). The relative abundance of *Acinetobacter* sp. *8I-beige, Acinetobacter johnsonii, Acinetobacter idrijaensis*, and *Acinetobacter bereziniae* in the OD group were significantly increased, whereas *Aeromonas dhakensis, Chloroflexi bacterium*, and *Salmonella enterica* were decreased in the OD group compared to the NOD group.

A LEfSe analysis on microbiome data was performed to double-validate the study results. LEfSe analysis (**Figure 2B**) identified 44 significantly differential nasal microbial species between the OD and NOD groups (Wilcoxon rank-sum test, LDA values > 2, *P* < 0.05). Of these, *Acinetobacter johnsonii* was significantly enriched in the OD group, followed by *Prevotella sp* MGM2*, Sediminibacterium salmoneum, Asinibacterium sp* OR53, and *Flavobacterium spp* (LDA value > 3, *P* < 0.05). On the other side, *Mycoplasma arginini, Chlamydia abortus, Salmonella enterica*, and *Aeromonas dhakensis* were significantly enriched in the NOD group (LDA value > 3, *P* < 0.05). The compositional alteration of the nasal microbiota at different taxonomic levels was reported in **Supplementary Figure S1A**. Overall, the nasal microbiota composition of OD patients was distinctly different from that of NOD patients at different taxonomic levels.

To predict the nasal microbial functions, functional annotations of the raw reads were performed using the KEGG and carbohydrate-active enzymes (CAZy) databases. The KEGG analysis (**Supplementary Figure S1B**) showed that metabolism-associated pathways were predominant in CRS patients, including global and overview maps, carbohydrate metabolism, aa metabolism, and energy metabolism. Additionally, it was found that the other secondary metabolite biosynthesis-related genes were upregulated in the OD group, while energy metabolism and circulatory system-related genes were upregulated in the NOD group (**Figure 2C**). Furthermore, carbohydrate metabolism can be affected by altered carbohydrate-active enzymes (CAZymes) following microbiota dysbiosis (32). CAZy database function annotation showed that Glycoside Hydrolases (GHs), Glycosyl Transferases (GTs), and Carbohydrate Esterases (CEs) played crucial roles in our study (**Supplementary Figure S1C**), and taxon-function Circos plot demonstrated that *Staphylococcus* followed by *Caulobacter, Corynebacterium*, and *Acinetobacter* were the ones that mostly contributed to the alteration of the CAZymes (**Supplementary Figure S1D**).

### 3.4 Nasal Metabolomics of OD patients are distinct from those of NOD participants

**Figure 3:**
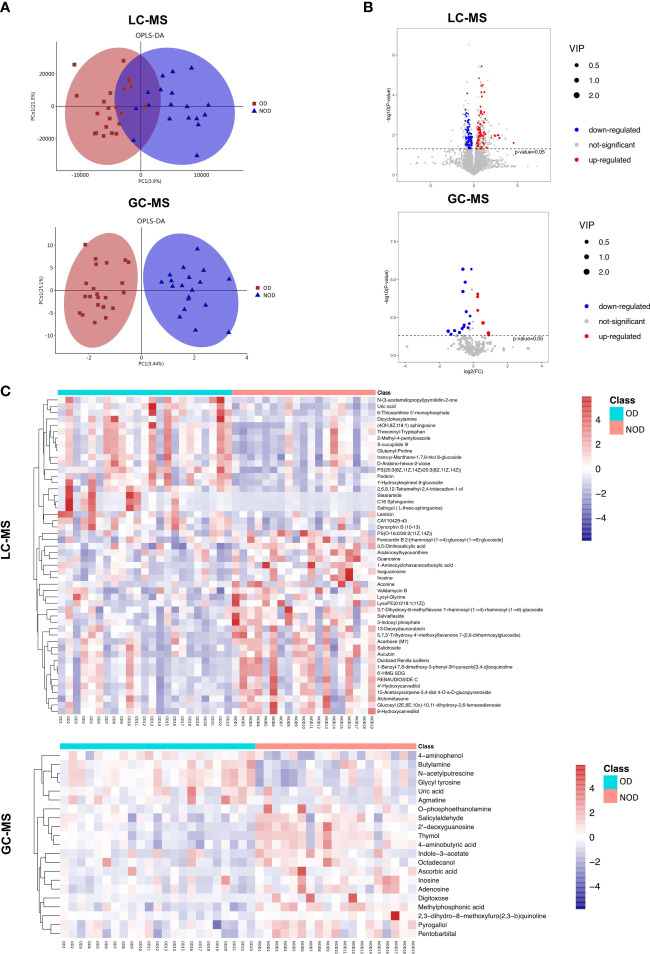
Characteristics of metabolites between OD (n = 23) and NOD patients (n = 19). **(A)** Orthogonal partial least squares discriminant analysis (OPLS-DA) plot of nasal metabolites of OD and NOD groups (above: LC-MS; below: GC-MS). **(B**, **C)** Volcano plot and hierarchical clustering heatmap of differential metabolites between OD and NOD groups (above: LC-MS; below: GC-MS). Heatmap showing individual metabolite levels of the samples (log-transformed). Red and blue shades represented high and low metabolite levels, respectively.

The OPLS-DA scores plot showed several differences in metabolomic profiles between the OD and NOD groups based on LC-MS and GC-MS analysis, which were further validated by permutation analyses (R^2^Y = 0.561, Q^2^Y = - 0.397; R^2^Y = 0.782, Q^2^Y = - 0.395, respectively) (**Figure 3A**). In LC-MS, the metabolome of OD patients reported 138 differential nasal metabolites compared to NOD patients, with increased and decreased levels of 69 and 69 metabolites, respectively as visualized in the volcano plot (*P* < 0.05, VIP > 1, **Figures 3B, C**). The most differential metabolites identified were: Salidroside, Alclometasone, Uric acid, trans-p-Menthane-1,7,8-triol 8-glucoside, Validamycin B, Salviaflaside, C16 Sphinganine, 1-Benzyl-7,8-dimethoxy-3-phenyl-3H-pyrazolo[3,4-c]isoquinoline, and Pederin, S-cucujolide III (**Supplementary Table S2**).

In GC-MS, the metabolome of OD patients reported 21 differential nasal metabolites in relation to NOD participants, with increased and decreased levels of 6 and 15 metabolites, respectively in OD patients (*P* < 0.05, VIP > 1, **Figures 3B, C**). The most differential metabolites identified were 4-aminophenol, Ascorbic acid, Uric acid, Thymol, 2’-deoxyguanosine, 2,3-dihydro-8-methoxyfuro(2,3-b)quinoline, 4-aminobutyric acid, O-phosphoethanolamine, Agmatine, and Inosine (**Supplementary Table S3**).

**Figure 4:**
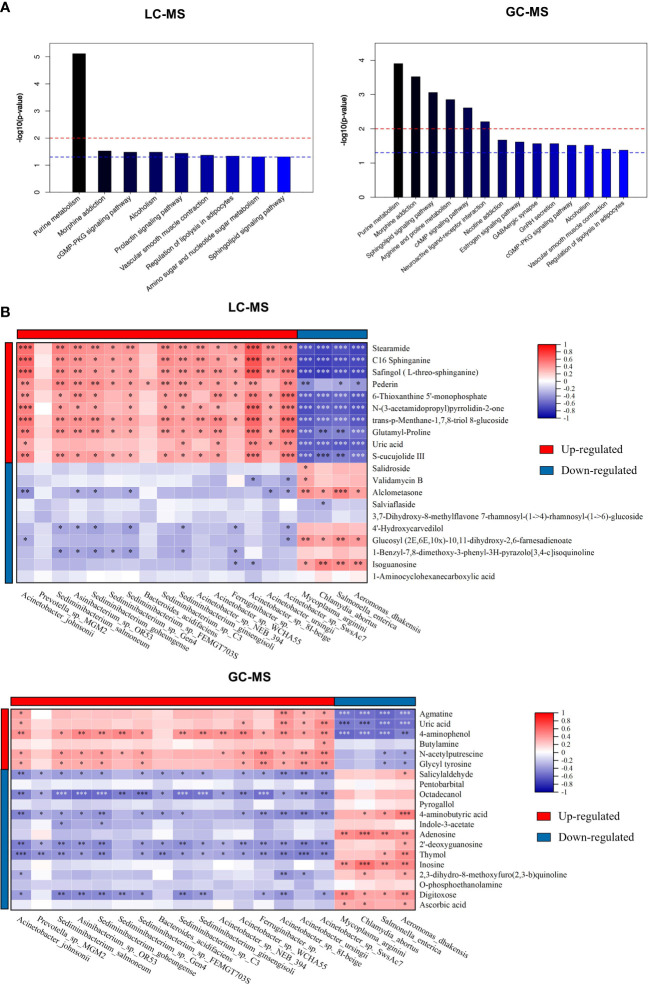
The metabolic pathway enrichment analysis of differential metabolites and correlation analyses of differential metabolites and altered microbial species. **(A)** Differential metabolic pathway using KEGG metabolic pathway enrichment analysis (left: LC-MS; right: GC-MS). **(B)** Spearman correlations between the 20 most differential nasal microbial species and 20 nasal metabolites presented as a heatmap (above: LC-MS; below: GC-MS). Red and blue squares indicated positive and negative correlations, respectively. *P < 0.05; **P < 0.01; *** *P* < 0.001.

Besides individual metabolites-based studies, the metabolic pathway enrichment analysis was used to investigate which metabolic sub-pathways play a significant role in olfactory dysfunction. KEGG pathway enrichment analysis demonstrated that the differential metabolites based on LC-MS and GC-MS were involved in purine, arginine and proline metabolism, morphine addiction, sphingolipid signaling and cAMP signaling pathways, and neuroactive ligand-receptor interaction (**Figure 4A**). Taken together, nasal metabolome analysis demonstrated that OD patients have distinct metabolites and metabolic pathways compared to NOD patients.

### 3.5 Correlation analysis between altered microbiota and differential metabolites identified

To identify the microbiota-metabolite interactions in the upper respiratory tract of CRS patients, the correlation between the relative abundance of altered nasal bacterial species and the levels of the differential metabolites identified was examined in relation to each other. As shown in **Figure 4B**, the heatmap built based on the Spearman correlations analysis indicated that differential metabolites were generally associated with the altered microbial species identified in both groups (for LC-MS and GC-MS, respectively, *P* < 0.05).

The metabolites enriched in OD-group, including trans-p-Menthane-1,7,8-triol 8-glucosidec, C16 Sphinganine, Pederin, S-cucujolide III, N-(3-acetamidopropyl)pyrrolidin-2-one, Glutamyl-Proline, 6-Thioxanthine 5’-monophosphate, Safingol (L-threo-sphinganine), and 2-Methyl-4-pentyloxazole, were positively correlated with specific OD-enriched microbes, including *Acinetobacter johnsonii, Acinetobacter* sp. 8I-beige, and *Acinetobacter* sp. NEB_394, but negatively correlated with certain OD-depleted microbes, such as *Aeromonas dhakensis, Chlamydia abortus, Mycoplasma arginini*, and *Salmonella enterica*.

On the contrary, specific OD-depleted metabolites, including Alclometasone, Glucosyl (2E,6E,10x)-10,11-dihydroxy-2,6-farnesadienoate, and Isoguanosine were in a strong positive correlation with OD-depleted microbes, as *Aeromonas dhakensis, Chlamydia abortus, Mycoplasma arginini*, and *Salmonella enterica*, but negatively correlated with certain OD-enriched microbes, such as *Acinetobacter johnsonii, Acinetobacter* sp. 8I-beige, and *Acinetobacter* sp. SwsAc7. Altogether, these findings confirmed the significant interactions between nasal inter-group differential metabolites and microbial species.

### 3.6 Alterations in the microbiome and metabolome between different severity of OD

**Figure 5:**
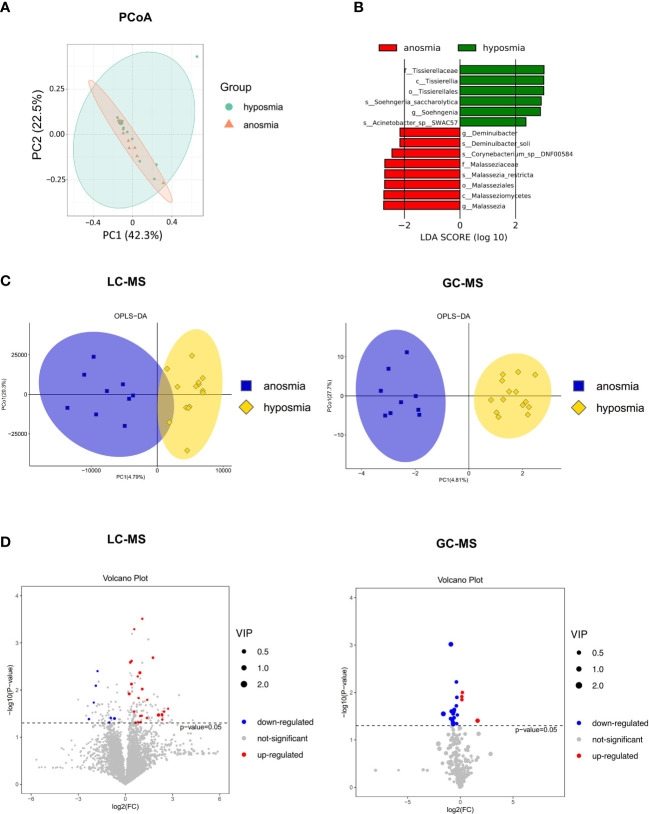
Differences in the microbiome and metabolome in relation to different severity of OD. **(A)** Principal coordinates (PCoA) analysis of nasal microbiota in hyposmia and anosmia groups. **(B)** Differential species in the hyposmia and anosmia groups based on LEfSe analysis. **(C)** Orthogonal partial least squares discriminant analysis (OPLS-DA) plot of nasal metabolites in hyposmia and anosmia groups (left: LC-MS; right: GC-MS). **(D)** Volcano plots of differential metabolites between the two groups (left: LC-MS; right: GC-MS).

Principal coordinates (PCoA) analysis based on Bray-Curtis dissimilarity demonstrated that there was not a significant difference in the overall nasal microbiota composition between patients with anosmia (absent olfactory function) and patients with hyposmia (reduced olfactory function) at a species level (PERMANOVA, R2 = 0.034, *P* = 0.69) (**Figure 5A**). LEfSe analysis identified 14 significantly differential nasal microbes between the anosmia and hyposmia groups (Wilcoxon rank-sum test, LDA values > 2, *P* < 0.05, **Figure 5B**). Of these, *Malassezia* and *Deminuibacter* genera were significantly enriched in the anosmia group with higher LDA values, indicating that they can be considered as potential biomarkers to predict severe olfactory disorders.

A clear demarcation of the nasal metabolite composition between patients with different OD severity was also obtained with the OPLS-DA analysis (**Figure 5C**). Indeed, in LC-MS, 7 and 26 metabolites were upregulated and downregulated, respectively, in the anosmia group as shown in the volcano plot and hierarchical clustering heatmap (*P* < 0.05, VIP > 1, **Figure 5D** and **Supplementary Figure S2A**). The top differentially abundant metabolites identified were Butabarbital, 9R,10S-Epoxy-3Z,6Z-eicosadiene, Irigenin, Dibenzyl ether, Cyclolinopeptide H, and beta-D-fructose 2,6-bisphosphate. In GC-MS, the levels of 4 and 17 metabolites were upregulated and downregulated, respectively in the anosmia group (*P* < 0.05, VIP > 1, **Figure 5D** and **Supplementary Figure S2B**). The most identified metabolites were Dehydroascorbic acid, 2,3-dihydro-8-methoxyfuro(2,3-b)quinoline, Homoserine, L-glutamine dehydrated, and L-valine. Therefore, patients with different severity of OD exhibited different nasal microbial compositions and metabolic output.

### 3.7 Nasal inflammatory mediators levels and their correlations with nasal microbiota, metabolites, and clinical disease indicators

**Figure 6:**
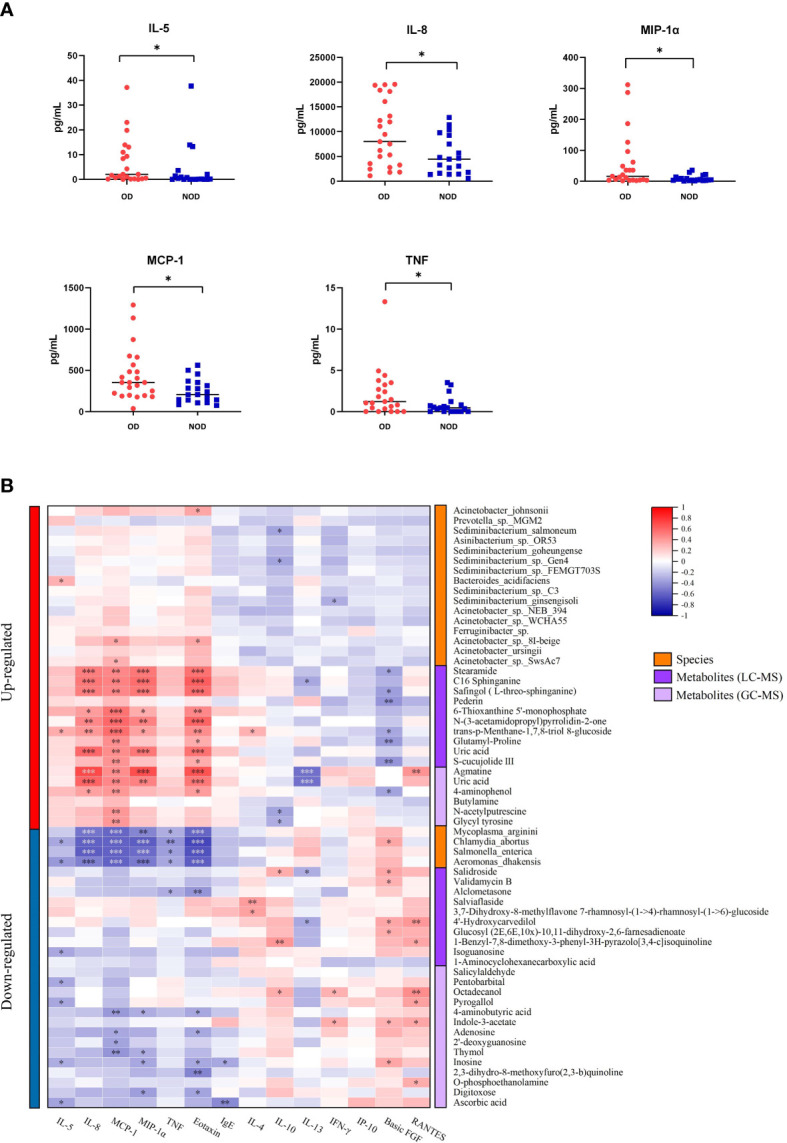
Correlation of nasal inflammatory cytokine levels with microbiota and metabolites. **(A)** Differences in nasal inflammatory cytokine levels between OD and NOD groups. **(B)** Spearman correlations between nasal cytokines, microbiota, and metabolites. Red and blue squares indicated positive and negative correlations, respectively. *P < 0.05; **P < 0.01; *** *P* < 0.001.

The levels of inflammatory mediators in the nasal mucus of OD and NOD participants were measured by CBA assays, showing that the levels of IL-5, IL-8, MIP-1α, MCP-1, and TNF were significantly higher in the OD group than in the NOD group (*P* = 0.030, *P* = 0.025, *P* = 0.031, *P* = 0.019, and *P* = 0.029, respectively, **Figure 6A** and **Supplementary Table S4**).

**Figure 7:**
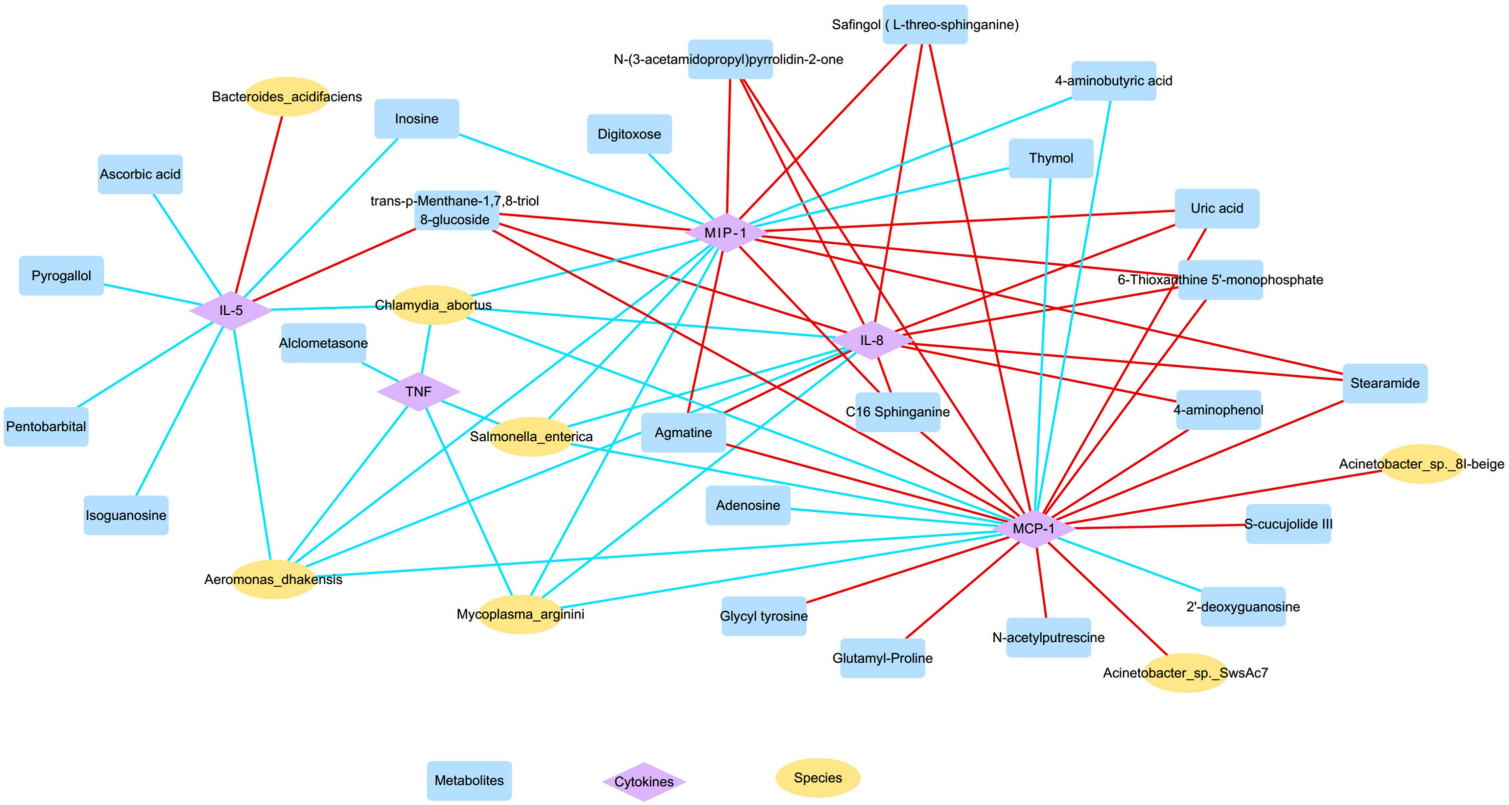
The microbiota-metabolites-cytokines interaction network diagram based on the altered nasal microbial species, metabolites, and inflammatory cytokines (yellow, blue, and purple nodes represent microbial species, metabolites, and inflammatory cytokines, respectively; red and blue lines represent positive and negative correlations, respectively).

The correlations between nasal microbiota, metabolites, and cytokines were reported in **Figure 6B** and **Figure 7**. The levels of several inflammatory mediators, including IL-5, IL-8, MIP-1α, MCP-1, and TNF, were positively correlated with OD-enriched microbial species, such as *Acinetobacter johnsonii, Acinetobacter* sp. 8I-beige*, Acinetobacter* sp. SwsAc7, and *Bacteroides acidifaciens*, and with OD-enriched metabolites, such as Uric acid, trans-p-Menthane-1,7,8-triol 8-glucoside, C16 Sphinganine, S-cucujolide III, N-(3-acetamidopropyl)pyrrolidin-2-one, Stearamide, Glutamyl-Proline, 6-Thioxanthine 5’-monophosphate, Safingol (L-threo-sphinganine). In contrast, these OD-specific cytokines were negatively correlated with OD-depleted microbes, including *Aeromonas dhakensis, Chlamydia abortus, Mycoplasma arginini, and Salmonella enterica*, and OD-depleted metabolites, including Alclometasone, Isoguanosine, Ascorbic acid, Thymol, 2’-deoxyguanosine, and 4-aminobutyric acid.

Furthermore, OD-enriched microbiota, metabolites, and cytokines showed positive correlations with clinical disease indicators of CRS, such as blood eosinophil and basophil percentages, Lund-Kennedy endoscopy scores, and Lund-Mackay CT scores, but negative correlation with blood neutrophil percentages (**Supplementary Figure S3**). On the other hand, OD-depleted species and metabolites showed opposite results, showing negative correlations with blood eosinophil and basophil percentages, endoscopy, and CT results, but positive correlations with blood neutrophil percentages. More importantly, these specific biomarkers revealed an intensive association with TDI scores, which are indicative of olfactory function. Taken together, the dysregulated nasal microbiota, metabolites, and inflammatory mediators showed clear associations with each other forming intensive interaction networks.

## 4 Discussion

In the current study, an integrated analysis of the metagenome, metabolome, and inflammatory mediators from the nasal mucus of CRS patients was performed to investigate their potential effects on olfactory function. Our findings demonstrated that nasal microbiota, metabolites, and inflammatory cytokines were consistently altered in OD patients compared to NOD patients, suggesting that nasal microenvironmental disorders may increase the risk of susceptibility to OD in CRS patients. Intensive correlations between the altered nasal microbiota, dysregulated metabolites, and over-expressive cytokines in OD patients were also found, suggesting that nasal microbial homeostasis might play crucial roles in the maintenance of the normal mucosal physiological function through the interaction with related metabolites and inflammatory mediators.

Accordingly with previous reports (10, 11, 33), our results demonstrated increased elevated levels of inflammatory cytokines (including IL-5, IL-8, MIP-1α, MCP-1, and TNF) in the nasal mucus of OD patients, indicating a disturbed mucosal immune function of OD patients. IL-5 is the crucial inflammatory cytokine that mediates the eosinophilic inflammation of CRS and directly contributes to the damage to olfactory neurons by releasing eosinophil-derived neurotoxins (12, 34). *Rouyar et al.* also demonstrated that immature olfactory neurons were decreased in the presence of prolonged type 2 inflammation which is characterized by an increased level of IL-5, indicating the inhibition of turnover of the olfactory neurons (8). In addition, IL-8 was significantly elevated in OD patients suggesting the presence of neutrophilic inflammation. Recent studies also revealed that the generalized neutrophilic inflammation in the nasal cavity is implicated in decreased levels of olfactory sensory neurons within the olfactory epithelium and the olfactory bulb and that the neutrophil recruitment mediated by IL-8 may disrupt the nasal epithelial barrier, inducing tissue remodeling, and further invoking an accumulation of eosinophils (35–37). On the other side, the Charcot-Leyden crystal, which is a representative product of eosinophils, was found to facilitate neutrophil chemotaxis by inducing the release of IL-8 (38). MIP-1α and MCP-1 are chemokines that regulate cell migration playing key roles in type 2 inflammation response. Specifically, MIP-1α, which is produced by a variety of inflammatory cells, induces a pronounced chemotactic effect on eosinophils in order to stimulate T-cells and regulate the IgE production (39, 40), while MCP-1 is able to attract monocytes and eosinophils, activate basophils and mast cells, and elicit leukotriene-C4 release leading to airway hyperreactivity and remodeling (41). Additionally, TNF-α is a typical inflammatory cytokine involved in type 1 immune response against bacterial infections and plays a crucial role in OD pathogenesis by mediating the progressive loss and regenerative inhibition of olfactory sensory neurons (42). Our results showed that mixed eosinophilic-neutrophilic inflammation and dysregulated type 1/type 2 immune response are both implicated in the pathogenesis of OD in CRS patients. However, the specific interactions However, the specific interactions of the inflammatory responses with olfactory function still remain unclear and require further investigation still remain unclear and require further investigation.

The shotgun metagenomics approach can provide a comprehensive picture of the microflora structure, enabling a more specific taxonomic classification and functional studies of microbial gene sequences in the environment than 16S rRNA gene-based amplicon sequencing (43). Our analysis on nasal metagenomic sequences revealed that OD patients showed a reduced microbial diversity and a significantly different nasal microbiota composition compared to NOD patients. *Fluitman et al.* reported that poor odor identification ability was significantly correlated with a decreased oral microbiota diversity in older adults, partially in line with the findings of our study (44). Nasal microbiota diversity alterations were also reported in relation to chronic rhinosinusitis, demonstrating that patients with eosinophilic CRSwNP showed a higher α-diversity compared with healthy controls (45). In general, hyposmia is associated with eosinophilia in CRS patients and therefore may also share a higher microbiota alpha-diversity, but this hypothesis was not confirmed in the present study. This may be due to the fact that the OD group in the present study reported predominantly a mixed eosinophilic-neutrophilic inflammation, which was corroborated in the inflammatory factor section of our study. Several microbial species were depleted in the OD group, such as *Mycoplasma arginini, Aeromonas dhakensis, and Salmonella enterica*, and negatively correlated with biomarkers of type 2 inflammatory response. Recent evidence suggested that specific microbes and microbial lysates may enhance the production of type 1 anti-inflammatory cytokines like IL-10 and IFN-γ, while inhibiting the overexpression of type 2 inflammatory factors like IL-4, IL-5, IL-13, and eotaxin, reversing the imbalance in type 1/type 2 immune response of allergic disease (46, 47). In this regard, *Mycoplasma arginini* is a bacterial specie that lacks a cell wall and it is implicated in modulating the host’s immune response (48). *M. arginini* could activate the substantial secretion of complement C3 from mesenchymal stem cells and inhibit Ig production by IL-4-induced B cells (49). Additionally, *Aeromonas dhakensis* is an autochthonous bacterium found in aquatic environments and is implicated in bacteremia and wound infections (50). *Fernández-Bravo* et al. reported that *A. dhakensis* induced the overexpression of TNF-α together with a variety of immune-related genes, which might in turn be responsible for moderating the overwhelming type 2 immune response, re-establishing the type 1/type 2 immune balance (51). Some studies have shown that *Salmonella enterica* and *Salmonella*-based oral multiantigen DNA vaccines could result in a shift towards type 1 immune responses, as well as the presence of high levels of IgA in the nasal mucosa (52, 53).

In addition, *Acinetobacter johnsonii* was significantly increased in OD patients and positively correlated with eotaxin, which is a type 2 inflammatory mediator involved in eosinophilic inflammation. Additionally, other OD-enriched species belonging to *Acinetobacter* genera, such as *Acinetobacter* sp. 8I-beige and *Acinetobacter* sp. SwsAc7, also showed similar associations with eosinophilic factors in our study, including eotaxin and MCP-1. It is well known that *Acinetobacter* belongs to the *Proteobacteria* family and it is associated with neurodegenerative and autoimmune diseases (54). However, further studies will be needed to validate its role in the development of OD. Moreover, *Malassezia*, a genus of fungi consisting of lipid-dependent basidiomycetous yeasts, was significantly enriched in patients with anosmia and it could be considered a potential biomarker of severe OD. The role of *Malassezia* in rhinosinusitis was also reported by *Lee et. al*, who found that bacteria-*Malassezia* interactions in the nasal mucosa could exacerbate inflammatory and allergic reactions by enhancing the expression of fungal sensing receptor Dectin-1 together with IL-5, IL-13, and IL-17 (55). In addition, epigenetics play a crucial role in microbial-host immune interactions as some opportunistic chronic pathogens can interfere with host DNA expression through epigenetic modifications and host cells’ pro-inflammatory events such as IL-1β release might be influenced by DNA methylation or histone deacetylase action (56). It should be noted that our study is a cross-sectional correlational study and the alterations in the microbiome may also be due to disturbed immune responses in the nasal mucosa in patients with chronic rhinosinusitis. The impaired host immune homeostasis can affect normal bacterial phagocytosis through the release of particulate proteins and the generation of reactive oxygen species (ROS) to shape the microbiome (57). Therefore, the causal relationships between altered nasal immune responses and microbial disorders and their role in the pathophysiological mechanisms of OD need to be further explored in future laboratory studies.

Metabolomics is a bridge between genotype and phenotype and can reflect the final effect of combined factors. Indeed, effectively small changes in gene and protein expression can be amplified in metabolites and are more easily detected by metabolomic analysis, holding an important role in the diagnosis of disease (58). Our nasal metabolomics analysis showed that OD patients reported alteration in diverse nasal metabolites when compared to NOD patients. Of these, uric acid, which is a damage-associated molecular pattern (DAMP) molecule, was increased in the OD group, with the consequent result to stimulate the innate immune system to avoid microbial invasion and cell death (59). The injured epithelial cells may trigger the eosinophil migration and degranulation through the release of DAMPs and induce an eosinophilic inflammatory response (60). Thus, the increased uric acid may influence the pathophysiology of OD by activating eosinophilic inflammation within the olfactory epithelium. In addition, 3,7-Dihydroxy-8-methylflavone 7-rhamnosyl-(1->4)-rhamnosyl-(1->6)-glucoside, which is a decreased lipid-like metabolite in the OD group belonging to flavones and flavonols categories, was associated with anti-inflammatory, anti-allergic, and neuroprotective properties (61). Increasing evidence suggests that flavonoids could ameliorate allergic inflammation by suppressing IL-4 and IL-13 production following the release of basophils and histamine (62), and improve the cognitive impairment of patients with neurodegenerative disease by inhibiting TNF-α and IL-6 release (63, 64). Furthermore, Thymol, which is also decreased in the OD group, showed potent anti-bacterial and anti-inflammatory properties in several studies. Thymol could indeed disrupt the mature bacterial biofilm, suppress the biofilm formation, and eliminate the *Staphylococcus aureus* infection (65). Additionally, thymol also showed a remarkable effect on relieving airway inflammation in allergic rhinitis and asthma by inhibiting type 2 cytokines, IgE expression, and the activation of the NF-κB signaling pathway (66, 67). The indole-3-acetate (IAA), which is a tryptophan-derived bacterial metabolite, was depleted in OD patients in this study. *Krishnan et al.* reported that IAA may attenuate the over-expression of several pro-inflammatory mediators and the corresponding genes in macrophages, including TNF-α, IL-1β, and MCP-1 (68). Moreover, a decreased IAA level was also reported in the chronic depression mouse model (69). It is well known that depression and OD are linked as they share the same anatomical basis, including the hippocampus, amygdala, and anterior angular cortex, suggesting a potential role of the reduced IAA level in the pathogenesis of OD (70). The pathway enrichment analysis performed in our study indicated a significant alteration of the purine metabolism pathway in nasal mucus samples in OD patients compared to NOD patients. In this regard, purines play a key role in cell growth-related signaling, neuromodulation, and neurotransmission, as well as energy metabolism (71). The role of purine metabolism in OD has been reported in the previous study, indicating that purine and lipids metabolomic disturbance was involved in the olfactory bulb dysfunction of depressive rats (72). Therefore, the final metabolite of purines, uric acid, as mentioned above, may be involved in eosinophilic inflammation and contribute to olfactory disorder in patients with CRS.

By integrating the correlation analysis of multiple omics data in this study, such as metagenome, metabolome, and inflammatory mediator arrays, a clear interaction network of the altered nasal flora, dysregulated metabolites, and over-expressive inflammatory cytokines was demonstrated, in accordance with recent studies. *Sun et al.* found that the microbiota-derived metabolite indole is able to suppress the formation of NLRP3 inflammasomes and the activation of the NF-κB signaling pathway while attenuating the neuroinflammation in Alzheimer’s disease (AD) mice (73). Additionally, *Yan et al.* also reported that the altered gut microbiota compositions during Parkinson’s disease (PD) could result in the decline of peripheral branched-chain amino acids (BCAAs) levels and that a high BCAA diet could reduce the inflammatory responses, reverse the dopaminergic neuron damage, and improve motor and non-motor dysfunctions in PD mice (74). Besides, microbiota-derived short-chain fatty acids (SCFAs) could activate the STAT3, mTOR, and ERK1/2 pathways, upregulate the transcription factor B lymphocyte-induced maturation protein 1 (Blimp-1) and promote the anti-inflammatory cytokine IL-10 to suppress the progression of inflammatory bowel disease induced by pathogenic T helper type 1 (Th1) cells (75). However, it should be pointed out that the role of specific microbiota-metabolites-immune interaction networks in the pathogenesis of OD still remains unclear and further investigations are needed.

Several limitations are in the current study. First, the sampling size was relatively small due to limited conditions, therefore the evaluation of key nasal biomarkers should be assessed in a larger study in the future. Second, although our results provided a picture of nasal microbiota-metabolites-immune interaction networks, the underlying mechanisms require further investigations with animal experiments and intervention studies. Third, the current study represents a preliminary investigation into the role of microbiota-metabolite-immune interaction in the occurrence and development of CRS-related OD, and therefore the effects of different CRS endotypes on olfactory functions should be further explored given the heterogeneity of CRS.

## 5 Conclusion

In conclusion, our results identified key microbial species, metabolic outputs, and inflammatory cytokines that may affect the olfactory function of CRS patients, and the distributed microbiota-metabolites-immune interaction network in OD patients was described. These findings provide insightful insights into our understanding of the role of nasal microbiota and associated metabolites in OD pathogenesis, however, the underlying mechanisms should be the object of further investigations.

## Data availability statement

The data presented in the study are deposited in the NCBI BioProject repository, accession number PRJNA949277; and MetaboLights repository, accession number MTBLS7584.

## Ethics statement

The studies involving human participants were reviewed and approved by the ethics committee of Beijing Anzhen Hospital (GZR-3-077). The patients/participants provided their written informed consent to participate in this study.

## Author contributions

XHa, ZW, and SW contributed to the conception and design of the study. Xingyu Han organized the database. Xingyu Han conducted the statistical analysis. XHa drafted the manuscript. XHa, XHe, XZ, LY, ZS, XG, SW, and ZW reviewed and edited the manuscript. All authors contributed to the article and approved the submitted version.

## Supplementary material

Supplementary Figure 1 Taxonomic differences of nasal microbiota between OD and NOD group and gene function annotation. **(A)** Compositional alteration of the nasal microbiota at different taxonomic levels based on LEfSe analysis. **(B)** KEGG function annotation of the nasal microbiota. **(C)** CAZy function annotation of the corresponding genes. **(D)** The taxon-function Circos plot.

Supplementary Figure 2 Differential metabolites between hyposmia and anosmia groups. **(A, B)** The hierarchical clustering heatmap of nasal differential metabolites between the hyposmia and anosmia groups (**A**: LC-MS; **B**: GC-MS). Heatmap showing the individual metabolite levels in the samples (log-transformed). Red and blue shades represented high and low metabolite levels, respectively (see color scale).

Supplementary Figure 3 Spearman correlations between levels of nasal cytokines, microbiota, metabolites, and clinical disease indicators. Red and blue squares indicate positive and negative correlations, respectively. **P* < 0.05; ***P* < 0.01; *** *P* < 0.001.

Supplementary Table 1 Differential microbial species between OD and NOD groups based on the Metastats analysis.

Supplementary Table 2 Differential metabolites based on LC-MS analysis between OD and NOD groups.

Supplementary Table 3 Differential metabolites based on GC-MS analysis between OD and NOD groups.

Supplementary Table 4 The inflammatory cytokines level comparison between OD and NOD groups.
