# Supplementary figures and images for: Disturbed microbiota-metabolites-immune interaction network is associated with olfactory dysfunction in patients with chronic rhinosinusitis
Source: Front Immunol. 2023 May 23;14:1159112. doi: 10.3389/fimmu.2023.1159112 (PMC10245275; doi:10.3389/fimmu.2023.1159112)

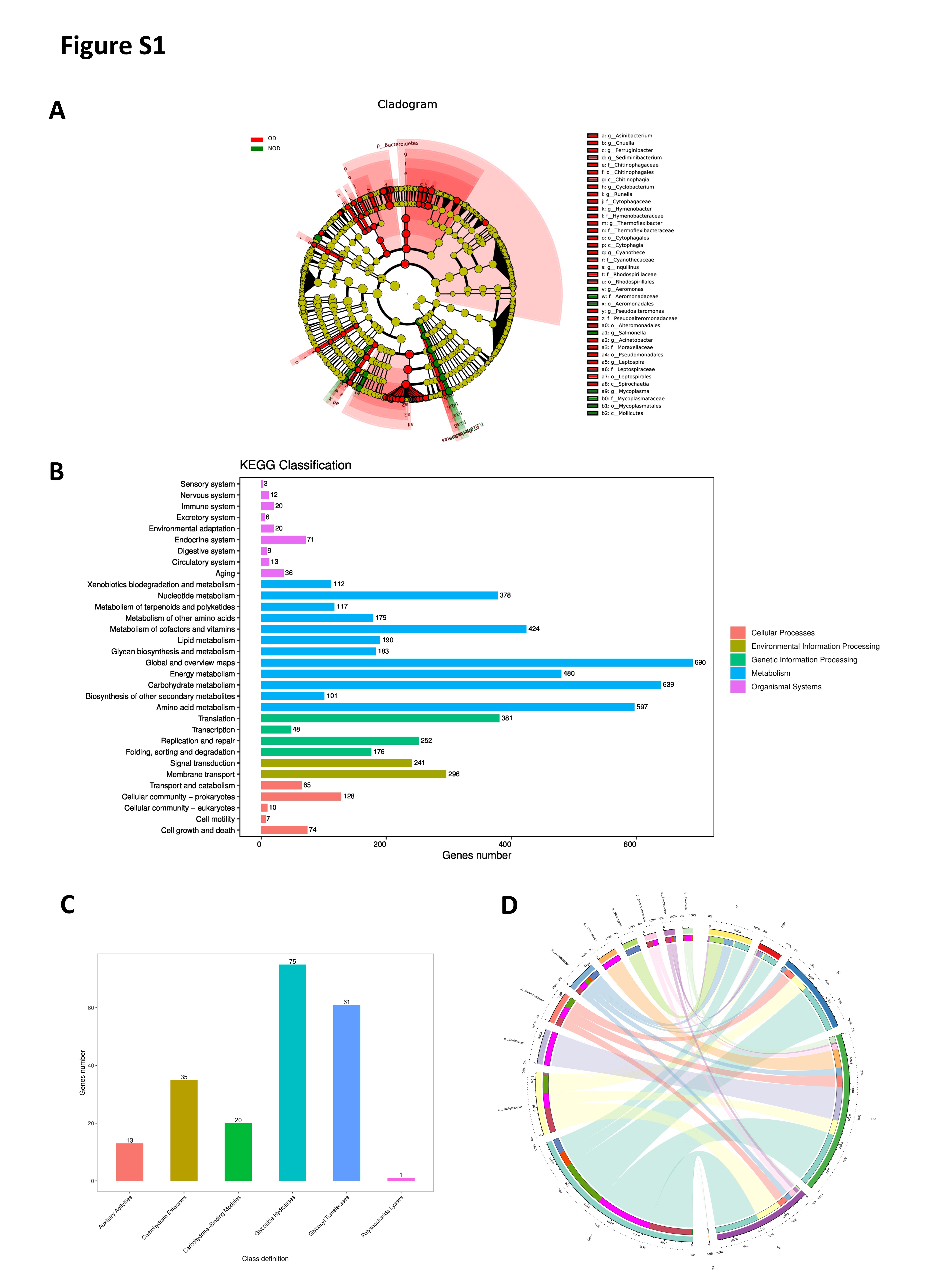

Supplement: Supplementary Figure 1 — Taxonomic differences of nasal microbiota between OD and NOD group and gene function annotation. (A) Compositional alteration of the nasal microbiota at different taxonomic levels based on LEfSe analysis. (B) KEGG function annotation of the nasal microbiota. (C) CAZy function annotation of the corresponding genes. (D) The taxon-function Circos plot. [file Image_1.jpeg]

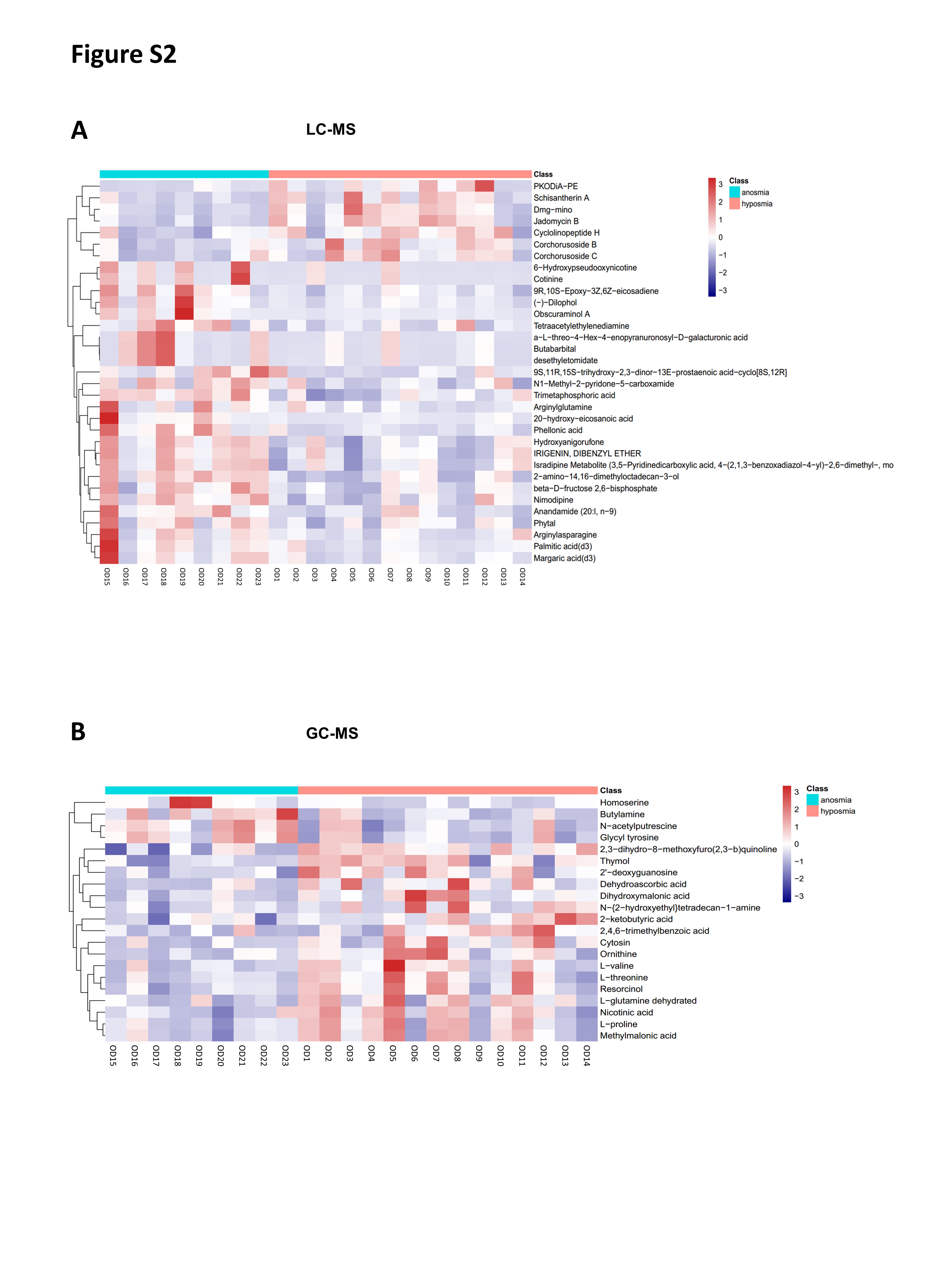

Supplement: Supplementary Figure 2 — Differential metabolites between hyposmia and anosmia groups. (A, B) The hierarchical clustering heatmap of nasal differential metabolites between the hyposmia and anosmia groups (A: LC-MS; B: GC-MS). Heatmap showing the individual metabolite levels in the samples (log-transformed). Red and blue shades represented high and low metabolite levels, respectively (see color scale). [file Image_2.jpeg]

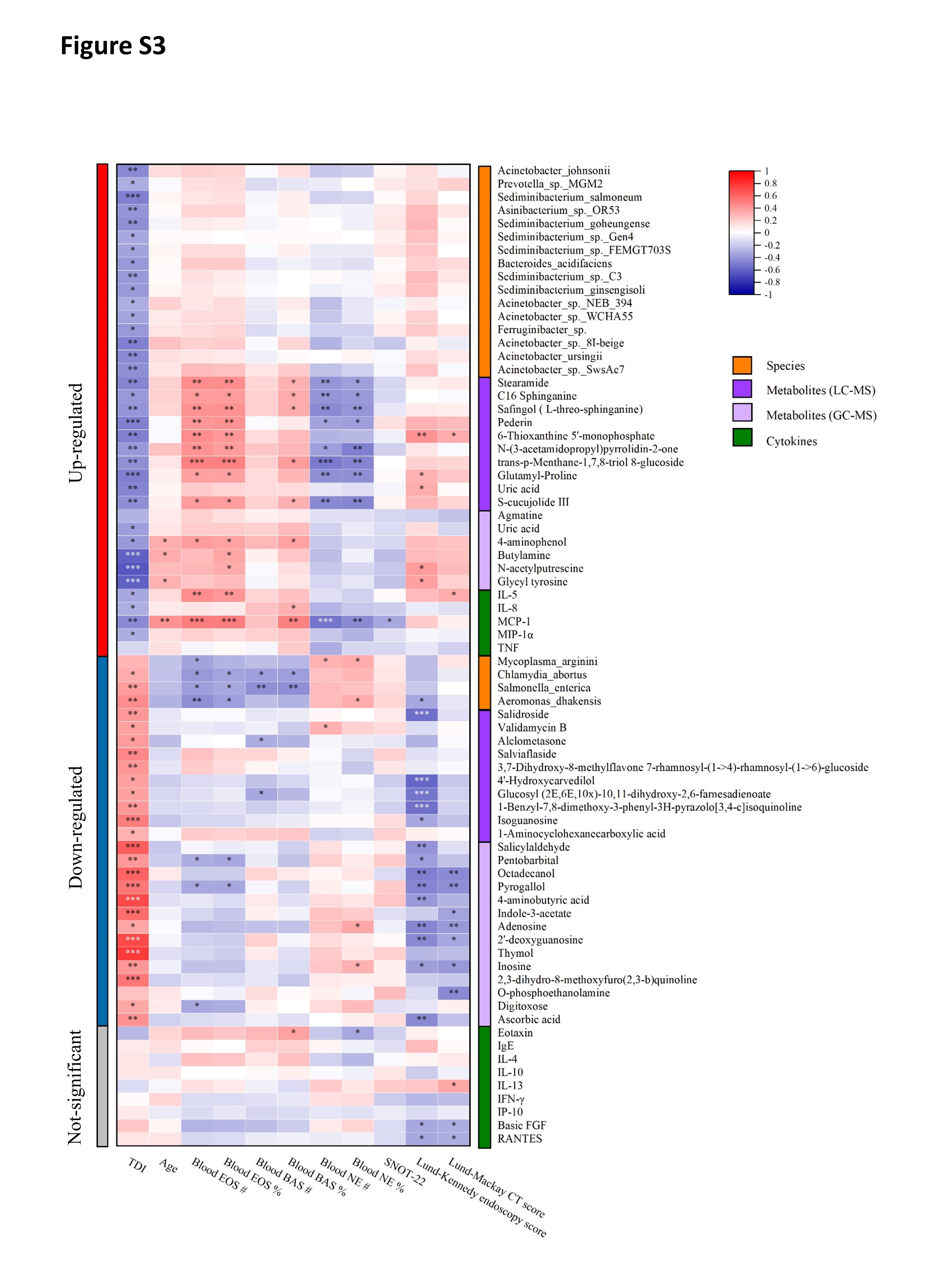

Supplement: Supplementary Figure 3 — Spearman correlations between levels of nasal cytokines, microbiota, metabolites, and clinical disease indicators. Red and blue squares indicate positive and negative correlations, respectively. *P < 0.05; **P < 0.01; *** P < 0.001. [file Image_3.jpeg]
